# Whole genome sequences of *Yersinia pestis* strains of ancient phylogenetic branch 0.ANT5 isolated in the 21st century in the Tien-Shan in Kyrgyzstan

**DOI:** 10.1128/mra.00469-24

**Published:** 2024-08-29

**Authors:** Alina N. Balykova, Alexander D. Katyshev, Galina A. Eroshenko, Lyubov M. Kukleva, Aigul K. Dzhaparova, Ekaterina A. Naryshkina, Eugene G. Oglodin, Stalbek K. Berdiev, Vladimir V. Kutyrev

**Affiliations:** 1 Russian Research Anti-Plague Institute “Microbe”, Federal Service for Surveillance in the Sphere of Consumers Rights Protection and Human Welfare, Saratov, Russia; 2 Republican Center of Quarantine and Particularly Dangerous Infections of the Ministry of Health of the Kyrgyz Republic, Bishkek, Kyrgyz Republic; University of Maryland School of Medicine, Baltimore, Maryland, USA

**Keywords:** *Yersinia pestis*, genomics, plague, molecular epidemiology, evolution, phylogenetic analysis

## Abstract

We announce the whole genome hybrid sequences of 11 highly virulent *Yersinia pestis* strains of the ancient phylogenetic branch 0.ANT5, one of the closest to the strains of the First Plague Pandemic. Nine strains were isolated in 2013–2023 and two in 1953 and 1971 in the Tien Shan plague focus in Kyrgyzstan.

## ANNOUNCEMENT

*Yersinia pestis* strains of the ancient phylogenetic branch 0.ANT5, one of the closest to the First Plague Pandemic strains (1–4), are widespread in the Tien Shan high-mountain focus in Kyrgyzstan. In this work, we obtained hybrid whole genome assemblies of 11 *Y. pestis* strains isolated in the Tien Shan high-mountain plague focus in 1952, 1971, and 2013–2023 (Table 1). All strains in this work were obtained from the collection of the Republican Center of Quarantine and Particularly Dangerous Infections of the Ministry of Health of the Kyrgyz Republic.

**TABLE 1:** Accession numbers and basic statistics of MGI and nanopore reads of *Y. pestis* strains

Strain	Isolation source, date	Isolation place	SRA accession no. MGI/ONT	Data for MGI reads		Data for nanopore MinION reads			GenBank accession no. of complete hybrid assembly genome size
				Total no. of filtered read pairs	Coverage (x)	Total no. of filtered reads	ONT read N50 (bp)	Coverage (x)	
5M	Oropsylla silantiewi, 14.06.1952	Upper-Naryn high-mountain plague focus, Dzhety-Oguz Syrts	SRR28624261/SRR28624260	2,347,574	78	68,149K	24,372	207	GCA_039640435.1 4.7 Mb
A1694	Fleas, 07.07.1971	Upper-Naryn high-mountain plague focus	SRR28623939/SRR28623938	2,012,544	66	43,291K	26,786	130	GCA_039640405.1 4.7 Mb
8	Human, 27.08.2013	Sarydzhas high mountain plague focus, Issyk-Kul region	SRR28607551/SRR28607550	2,346,044	78	83,201K	15,493	181	GCA_039640455.1 4.8 Mb
2	Fleas, 28.06.2016	Sarydzhas high mountain plague focus, Issyk-Kul region	SRR28607219/SRR28607218	2,344,934	78	81,861K	20,745	222	GCA_039640445.1 4.8 Mb
197–15	Marmota, 16.07.2015	Upper-Naryn high-mountain plague focus, Ak-Shyyrak section	SRR28622503/SRR28622502	2,063,450	88	19,752K	28,815	67	GCA_039640465.1 4.7 Mb
177	Marmota baibacina, 08.09.2019	Upper-Naryn high-mountain plague focus, Karakol Department, Batyr-Beshik area	SRR28622483/SRR28622482	2,342,188	78	25,555K	23,092	77	GCA_039640475.1 4.7 Mb
80	Oropsylla silantiewi, 26.08.2020	Sarydzhaz high-mountain plague focus, Ak-Su district, Kooylu river basin, Oroysuu area	SRR28623134/SRR28623133	2,339,355	77	263,065K	11,016	460	GCA_039640425.1 4.8 Mb
91_KR	Oropsylla silantiewi, 19.08.2020	Sarydzhaz high-mountain plague focus, Ak-Su district, Kooylu river basin, Bordu-Tor area	SRR28623332/SRR28623331	2,352,174	78	47,836K	35,949	185	GCA_039640415.1 4.8 Mb
107	Marmota baibacina , May 2023	Sarydzhaz high-mountain plague focus, Chon-Karabel area	SRR28639070/SRR28639069	2,349,018	78	27,580K	11,211	46	GCA_039640485.1 4.8 Mb
136	Ochotona, May 2023	Sarydzhaz high-mountain plague focus, Chon-Karabel area	SRR28624414/SRR28624413	2,346,198	78	84,342K	9668	129	GCA_039640395.1 4.8 Mb
133	Marmota baibacina, May 2023	Sarydzhaz high-mountain plague focus, Chon-Karabel area	SRR28638969/SRR28638968	2,351,922	78	80,058K	10,905	134	GCA_039640175.1 4.8 Mb

Strains were grown from single colonies on LB agar (pH 7.2) for 24 h at 28°C. For DNA extraction, strains were grown in LB broth (pH 7.2) for 24 h at 28°C. The DNA of three strains (107, 133, and 136) was extracted using the PureLink kit (Invitrogen, USA). The DNA of the remaining strains was obtained using phenol−chloroform extraction (5). MGIEasy FS kit and the MGIEasy UDB PF Adaptor Kit A were used to generate 150-bp paired-end reads. The pooled libraries were sequenced on the MGI platform (DNBSEQ-G50RS) according to the manufacturer’s instructions. Libraries for sequencing long reads were prepared using a V14 ligation kit (SQK-LSK114; Oxford Nanopore Technologies [ONT], UK) with Native Barcoding Kit 24 v14 (SQK-NBD114.24). DNA was size selected using AMPure XP beads (Beckman Coulter, Inc.) in volume ratio 1: 0.4 sample to the beads but was not sheared. The pooled libraries were sequenced on FLO-PRO114M (R10.4.1) for 48 h according to the manufacturer’s instructions.

We removed adapters and verified the MGI read quality using fastp v0.23.3 (6). The “base-calling” stage was carried out using Oxford Nanopore Dorado v0.5.2 (https://github.com/nanoporetech/dorado) with dna_r10.4.1_e8.2_400bps_sup@v4.2.0 model. Reads were filtered using Filtlong v0.2.1 (Q score, >8; length, >2,000  bp) (https://github.com/rrwick/Filtlong). Trycycler v0.5.5 (including Flye v2.9.3) was used to generate consensus hybrid assemblies; Polypolish v0.6.0 was applied to polish genome assemblies with short reads (7–9). The genome was manually trimmed and reoriented to *dnaA*, the plasmids were reoriented to *repA* with Trycycler v0.5.5 (7). The assembly quality was assessed using QUAST v.4.3 (10) and Bandage v.0.8.1 (11). Default parameters were used for all software. The average genome size was 4.5 Mb, the median coverage of the resulting genomes was 134×, and the GC content was 47.5%. All strains contained three plasmids (pMT, pCD, and pPCP). The presence of plasmids in the genome was further confirmed by detecting plasmid DNA bands: ~96, 2 bp (pMT), ~70.3 bp (pCD), and ~9.6 bp (pPCP) using the method of C.I. Kado and S.T. Liu (12). Finally, the genomes were annotated using the NCBI Prokaryotic Genome Annotation Pipeline (PGAP) (13).

The obtained whole-genome hybrid sequences of *Y. pestis* strains of the phylogenetic branch 0.ANT5 are important for understanding the modern and historical directions of the evolution of pandemic strains of plague pathogen.

## Data Availability

The hybrid genome sequences, the raw long and short reads are deposited into NCBI GenBank under BioProject accession number PRJNA1098581 and are available under the accession numbers listed in Table 1.
